# Using Temporal Covariance of Motion and Geometric Features via Boosting for Human Fall Detection

**DOI:** 10.3390/s18061918

**Published:** 2018-06-12

**Authors:** Syed Farooq Ali, Reamsha Khan, Arif Mahmood, Malik Tahir Hassan, Moongu Jeon

**Affiliations:** 1 Department of Software Engineering, University of Management and Technology, UMT Road, C-II Johar Town, Lahore 54000, Pakistan; farooq.ali@umt.edu.pk (S.F.A.); reamshakhan1122@gmail.com (R.K.); tahir.hassan@umt.edu.pk (M.T.H.); 2 Department of Computer Science, Information Technology University (ITU), 346-B, Ferozepur Road, Lahore, Punjab 54000, Pakistan; arif.mahmood@itu.edu.pk; 3 School of Electrical Engineering and Computer Science, Gwangju Institute of Science and Technology (GIST), Gwangju 61005, Korea

**Keywords:** intelligent surveillance systems, human fall detection, health and well-being, safety and security

## Abstract

Fall induced damages are serious incidences for aged as well as young persons. A real-time automatic and accurate fall detection system can play a vital role in timely medication care which will ultimately help to decrease the damages and complications. In this paper, we propose a fast and more accurate real-time system which can detect people falling in videos captured by surveillance cameras. Novel temporal and spatial variance-based features are proposed which comprise the discriminatory motion, geometric orientation and location of the person. These features are used along with ensemble learning strategy of boosting with J48 and Adaboost classifiers. Experiments have been conducted on publicly available standard datasets including *Multiple Cameras Fall* (*with 2 classes and 3 classes*) and *UR Fall Detection* achieving percentage accuracies of 99.2, 99.25 and 99.0, respectively. Comparisons with nine state-of-the-art methods demonstrate the effectiveness of the proposed approach on both datasets.

## 1. Introduction

The increasing number of aged persons has led to the uncertainty of unaided and unprovoked falls which may cause physical harm, injuries and health deterioration. These problems may become more intense if timely aid and assistance is not available. To mitigate such effects and to control the risks, there must be an accurate fall detection system. For this reason, surveillance added technology for the timely and retrospective detection of falls has become a priority for the health care industry. Therefore, the development of an intelligent surveillance system is essential, specifically, a system which has the capacity to automatically detect fall incidences using surveillance cameras.

To cope with such a crucial need, various fall detection wearable devices have been developed [1]. Some devices contain buttons and sensors that can be pressed if there is an emergency [1]. However, these devices become ineffective or even useless if the subject is unable to press the button due to unconsciousness or being far from the device. Due to the failure of wearable devices, video controlling and monitoring systems have entered the arena [1], but these systems also suffer from inaccuracy and unreliability [1]. Generic action recognition systems such as [2] may not efficiently detect falls. Due to the lack of efficiency of wearable devices and generic video systems [3], it has become necessary for customized automatic fall detection systems to be developed to cope with the challenges posed by fall detection problems. Such systems could dramatically improve the health care of older people.

In the current paper, we propose a fall detection system based on the spatial and temporal variance of different discriminative features. The proposed system is compared with nine existing algorithms on two publicly available datasets. The proposed system has exhibited excellent performance on both datasets. It may be noted that, a video surveillance system may result in a privacy issues during the continuous monitoring of older adults, and thus, has its own limitations for individual home monitoring, but it could be useful for rehabilitation centers and elderly health care houses, reformation centers [4,5], nursing homes and hospitals, electronic care environments [6], disabled care centers [7], and firearm shot damage spotting centers [8].

The remainder of this paper is organized as follows. Section 2 contains related work, Section 3 contains the proposed system, experimental evaluations are given in Section 4, conclusions and future follow in Section 5.

## 2. Related Work

In recent years, several vision-based techniques have been proposed for human fall detection. Some of these are based on wearable devices, such as accelerometers, while others use depth sensors, such as Microsoft Kinect. Moreover, RGB video cameras and different types of audio sensors have also been used for fall detection. Depending upon the underlying hardware, algorithms have been proposed to use one or more modalities, including accelerometer output, depth data, audio data and/or video data. Following are the broad categories of fall detection approaches and techniques.

### 2.1. Classification Based on Input Data Types

#### 2.1.1. Sensor-Based

Luo et al. [9] developed a dynamic movement detection system to detect human falls. Their algorithm is based on the output of digital signals from mounted accelerometers. They filtered noisy segments using a Gaussian filter and set up a 3D body movement display which related different postures of the body to the yields of the accelerometers. Bourke et al. [10] described a procedure under supervised conditions to identify falls using tri-axial accelerometer sensors based on thresholding techniques. These sensors were mounted on the trunks and thighs of subjects. Makhlouf et al. [11] developed a multi-modal system that provided a fall detection service and an emergency service. Their system used photoelectric sensors and accelerometers to get information regarding the state of a person. If a person is in fall state, then the emergency service informs the doctor. The message sent to the specialist incorporates data about the localized area and condition of the individual. Recently, Casilari et al. [12] proposed a public repository of datasets that could be used as a common reference among the researchers to compare their algorithms. They created the UMAFall dataset that contains information about day-to-day activities and human falls. Contrary to other existing datasets that use one or two sensing points, they obtained data using five wearable sensors.

#### 2.1.2. Audio-Based

Zigel et al. [13] presented an automatic fall discovery framework for elderly individuals particularly for when a person is unconscious or tense. The framework depends on indoor vibration and sound detection to classify human falls and other events. The classification uses features that include the shock response spectrum and Mel-frequency cepstrum coefficients. Doukas et al. [14] proposed a fall detection system based on audio and video data. Tracking of a person was done using video, and sound directionality analysis was made from audio data. Various features, including acceleration, sound proximity, average peak frequency, average signal relative amplitude, visual blob size and average movement speed were trained and tested using an SVM classifier for the detection of falls. The post-fall analysis was conducted as well to further predict if a person recovered his state or remained unconscious.

#### 2.1.3. Image and Video-Based

Foroughi et al. [15] studied the morphological variations of silhouettes acquired from a series of videos. They concluded that the amalgamation of relative ellipse along the human body, the projection of histogram along the *x*-axis and *y*-axis and the change in the position of person’s head provided beneficial cues for the determination of various behaviors of falls. Miaou et al. [16] suggested that visual detection may cause false readings as sometimes a movement appears as a fall, but, actually it may not be a fall. It could be simply a motion towards the direction of ground.

Lee et al. [17] recognized falls using image-based sensors. The data was generated by asking the subjects to randomly repeat five scenarios including lying down in a ‘tucked’ position, lying down in a ‘stretched’ position, stooping, sitting/lying down in an inactive zone and walking/standing.

Rougier et al. [18] emphasized the use of computer vision techniques to provide promising solutions for the detection of human falls. They used human shape deformation in a video sequence for human fall detection. The shape deformation from the person’s silhouette was tracked along the video sequence. The fall was detected using a Gaussian mixture model. In [19], a vision-based system was proposed for human fall detection. The system used novel features of motion history for the detection of a fall. The system was run on video sequences of daily activities and simulated falls. Doulamis et al. [20] proposed a human fall detection system using cheap and low resolution cameras. Their system used adaptive background modeling using Gaussian Mixtures, and hierarchical motion estimation was used to distinguish falls from other activities, including lying, walking and sitting.

### 2.2. Classification Based on Classifier Types

#### 2.2.1. Thresholding-Based

Chariff et al. [21] proposed intelligent surveillance technology to be used for the detection of dangerous events in the home environment. They tracked individuals as ellipses, and the direction of motion was utilized to recognize irregular and abnormal activities. Tao et al. [22] described the use of activity summarization in supportive homes where care is provided to aged people, but the proposed fall detection system was not capable of differentiating between a real fall incident and when the subject was just lying down.

Zaid et al. [23] used mobile robots to provide an efficient solution for fall detection in elderly people. The mobile robot system used Kinect sensor to track a target person and detect when they had fallen. Moreover, in case of a fall, an alarm was generated by sending an SMS message notification or making an emergency call.

Sumiya et al. [24] proposed a versatile robot to recognize human falls and to give details to observers. It comprised of a family portable robot with Microsoft Kinect and a PC. For simplicity, a sensor was placed on the robot which limited the blind zone by moving around with the robot. This technique improved the accuracy of fall detection compared to monitoring techniques based on fixed sensors.

#### 2.2.2. Machine Learning-Based

Various classifiers including the Support Vector Machine (SVM), Adaboost, Multilayer Perceptron (MLP) and J48 have been used for human fall detection. Support Vector Machine, a pattern classification algorithm developed by V. Vapnik and his team at AT&T Bell Labs maps the data into higher dimensional input space and constructs an optimal plane separating the hyper-plane in this space. In [25], Foroughi et al. in 2008 implemented a Support Vector Machine to classify an event either as a fall or not a fall using feature-based approach and achieved a reliable recognition rate of 88.08%. Various other human fall detection systems use SVM for the classification of fall events (by their features) [26,27,28].

Debard et al. [26] proposed a feature-based approach for the detection of human falls. The proposed features included the angle of fall, aspect ratio, center velocity and head velocity. These features were trained and tested using SVM method. The drawbacks of this system include its inaccuracy to discriminate gradual fall from a person who is sitting down normally.

Ni et al. [29] developed a fall prevention framework for application in hospital wards. Their system detects if a patient gets up from the bed and generates an alarm for hospital staff to provide help. Their system used various features to detect human falls on a dataset of videos obtained from RGBD sensors of Microsoft Kinect. These included the region of interest (ROI), motion-based features, and shape-based features.

Adaboost [30], an adaptive boosting algorithm, utilizes a small number of weak classifiers that are used to construct cascades of strong classifiers. The combination of the strong classifiers into a cascade results in high accuracy and time efficiency for human fall detection. Multilayer Perceptron (MLP), a feedforward artificial neural network, is used for human fall detection systems, and achieved an accuracy of 90.15% on an ADL dataset [31].

The rule-based algorithm, J48, has also been used for the detection of human falls [32,33]. J48 is a C4.5 decision tree which is used to present different models of classification and also reveals human reasoning [34]. It has many advantages over various learning algorithms, such as its low computational cost of model generation, noise robustness and ability to handle redundant attributes and modules. It is robust even if training data contains errors or have missing attribute values [35]. Shi et al. proposed a human fall detection algorithm for classifying the human motion using the J48 decision tree classifier and achieved a sensitivity of 98.9%, a specificity of 98.5% and an overall accuracy of 98.6% [36]. In 2017, Guvensan et al. developed a system that implements the decision tree learning algorithm of J48, using five features, for detection of fall events [37]. Motivated by these algorithms, we chose the boosted J48 classifier due to its significantly higher F-measure, low computational cost and robustness to outliers and reduction of the feature space.

In the current work, we propose an algorithm under a boosting framework based on RGB video data for human fall detection. We emphasize its fast detection speed with high accuracy. We compare our work with existing state-of-the-art approaches, including Kepski et al. [38] by using KNN and SVM on the Multiple Cameras Fall Dataset [26] and the UR Fall Detection Dataset [38]. The results of our approach under an ensemble learning strategy with J48 and Adaboost outperform existing state-of-the-art approaches on both datasets in terms of percentage accuracy and execution time.

## 3. Proposed Fall Detection System

The first step in our proposed algorithm was to segment the foreground from the bac-ground and to identify the foreground as a person or non-person. In the second step, we computed various features from the foreground and in the third step, we trained a boosted J48 classifier for per frame classification of the foreground as a falling person or a stable moving person. In addition to the spatial information, our features also use temporal information as velocity and acceleration. Each of these steps is explained in the following sections.

### 3.1. Foreground Detection

A clean background image can be computed using the recently proposed background detection algorithms, such as [39,40,41]. The computed background image is subtracted from each frame to find the change region. Pixels with a change larger than a fixed percentage of the background image are considered to be , while the rest are considered to be the result of variation due to noise. In our experiments, we fixed this percentage at 15% of each pixel value in the background image. Then, a distance transform was computed over the changed region, followed by morphological operations, including erosion and flood filling to fill the holes in this region. To ensure that the change region contained only humans, connected components were computed and components with a size less than the minimum human size were deleted from the foreground. If a connected component had a size larger than the minimum person size threshold, it was considered to correspond to a human. Thus, a foreground mask containing only a human object was obtained. The minimum person size threshold helped to discard frames not containing a full person. Components with a size larger than the minimum person size threshold are referred to as foreground blobs in the rest of the paper.

### 3.2. Temporal and Spatial Variance for Falling Person Detection

**Figure 1:**
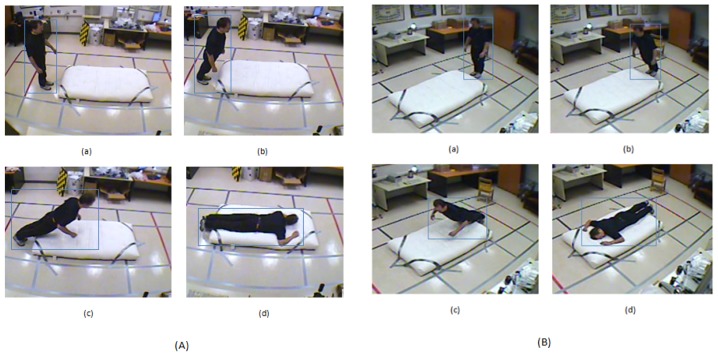
Temporal variation of the aspect ratio ($a_{r}$) of a falling person in the Multiple Camera Fall (MCF) dataset where $a_{r}$ = width/height of bounding box of a foreground blob. The person angle ($p_{a}$), shape deformation ($s_{d}$), and ellipse ratios ($e_{r}$) are also shown for each case. (**A**) Camera 7, video 1: (**a**) $a_{r}$ = 0.45, $p_{a}$ = 90${}^{{}^{\circ}}$, $s_{d}$ = 880.93, $e_{r}$ = 2.02, (**b**) $a_{r}$ = 0.48, $p_{a}$ = 68${}^{{}^{\circ}}$, $s_{d}$ = 670.35, $e_{r}$ = 2.17, (**c**) $a_{r}$ = 1.45, $p_{a}$ = 27${}^{{}^{\circ}}$, $s_{d}$ = 435.39, $e_{r}$ =1.85, (**d**) $a_{r}$ = 2.85, $p_{a}$ = 0${}^{{}^{\circ}}$, $s_{d}$ = 3252.91, $e_{r}$ = 0.43; (**B**) Camera 3, video 3, (**a**) $a_{r}$ = 0.44, $p_{a}$ = 90${}^{{}^{\circ}}$, $s_{d}$ = 580.99, $e_{r}$ = 2.12, (**b**) $a_{r}$ = 0.52, $p_{a}$ = 55${}^{{}^{\circ}}$, $s_{d}$ = 356.45, $e_{r}$ = 2.05, (**c**) $a_{r}$ = 1.46, $p_{a}$ = 27${}^{{}^{\circ}}$, $s_{d}$ = 193, $e_{r}$ = 1.42, (**d**) $a_{r}$ = 2.8, $p_{a}$ = 0${}^{{}^{\circ}}$, $s_{d}$ = 2152.48, $e_{r}$ = 0.38.

In this section we discuss various types of temporal variance that we used in the proposed real-time person falling detection system. These variances include temporal variations of the aspect ratio, fall angle, speed, upper half area of bounding box and geometric center of the connected component. The temporal variance for each parameter was computed over a temporal window of size k=30. This value of *k* was chosen after analyzing the video data and carefully conducting experiments. The value of k=30 corresponded to a duration of 1 s as the frame rate of the videos was 30 frames per second. Figure 1 shows variation of the aspect ratio and other parameters with a change in person position during the process of fall.

#### 3.2.1. Temporal Variance of the Aspect Ratio

A bounding box was computed containing the foreground blob. The aspect ratio refers to the ratio of the width to the height of this bounding box. The temporal variation of the aspect ratio is unique during the fall of a person, which was used as a feature. For a person in a stable position, the temporal variation in the aspect ratio is small, while during a fall, this variation is large. The temporal variation of the aspect ratio in a current frame was computed by taking the standard deviation of the aspect ratios of the previous *k* frames. After analyzing the video data and conducting experiments, the value of k=30 was found to be reasonable to capture temporal variations in this parameter.

(1) $$\sigma_{ar}^{2}=\frac{1}{k}\sum_{i=1}^{k}{\left(a_{r}\left(i\right)-\mu_{ar}\right)}^{2}$$

#### 3.2.2. Temporal Variation of the Person Angle

An ellipse was fitted in the foreground blob and a person angle was computed as the angle between the major axis of ellipse and the *x*-axis (horizontal axis). The person angle changed when a person fell from a standing state to a fall state. The temporal variation in the fall angle of the current frame was computed by computing the standard deviation of the fall angle of the previous k=30 frames.

(2) $$\sigma_{pa}^{2}=\frac{1}{k}\sum_{i=1}^{k}{\left(p_{a}\left(i\right)-\mu_{pa}\right)}^{2}$$

#### 3.2.3. Temporal Variation of the Motion Vector

**Figure 2:**
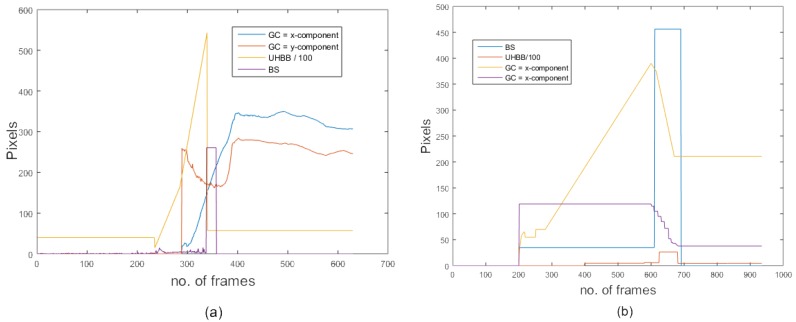
The temporal variation in body speed (B.S.), upper half area of the bounding box (U.H.B.B), and *x*-component and *y*-component of the geometric centre (G.C.) of a falling person on the MCF dataset: (**a**) camera 2, video 2; (**b**) camera 3, video 3.

The motion vector is the variation in the foreground blob’s position between the current and the next frame. The motion magnitude increases when the body is in motion, and it reaches a high value during fall and then it becomes zero after the fall. The change in the magnitude of the body speed (B.S.) serves as an important parameter for human fall detection. The body speed is calculated by computing the motion vectors of the centroid of the foreground blob. The magnitude of motion vectors is calculated as shown in Equation (3).

(3) $$|m_{v}|=\sqrt{{\left(m_{v}\left(x\right)\right)}^{2}+{\left(m_{v}\left(y\right)\right)}^{2}},$$

where $m_{v}\left(x\right)$ is the magnitude of motion vectors along the *x*-direction and $m_{v}\left(y\right)$ is the magnitude of motion vectors along the *y*-direction. The temporal variation in body speed gives the acceleration of the body. Figure 2 shows the body speed variation of a falling person where the number of frames and magnitude of motion vectors are plotted along *x*-axis and *y*-axis respectively. The temporal variance of $m_{v}$ is computed as follows:

(4) $$\sigma_{mv}^{2}=\frac{1}{k}\sum_{i=1}^{k}{\left(m_{v}\left(i\right)-\mu_{mv}\right)}^{2},$$

where $\mu_{mv}$ is the mean motion vector over the current time window.

#### 3.2.4. Temporal Variation of Shape Deformations

The orientation of the body shape changes significantly when a person falls. The upper half area of the foreground blob bounding box (U.H.B.B.) was used as a shape descriptor ($s_{d}$) to capture variations in the orientation of the body when fall occurs. The bounding box is divided into two equal halves. The upper half area of the upper half bounding box is high when a person is in fall state as compared to standing state. Hence, the area of the upper half of the bounding box serves as a strong feature as its value changes significantly when a person enters into a fall state from a normal state. Figure 2 shows the temporal variation of the upper half area of the bounding box of a falling person of video 1 (camera position 7) and video 3 (camera position 3), respectively. In these figures, the number of frames and number of pixels in the upper half area of bounding box are plotted along the *x*-axis and *y*-axis, respectively. The temporal variation in the upper half area of the bounding box of a current frame is computed by calculating the standard deviation in the upper half area of the bounding box from the previous k=30 frames to detect if an event is a fall or not a fall.

(5) $$\sigma_{sd}^{2}=\frac{1}{k}\sum_{i=1}^{k}{\left(s_{d}\left(i\right)-\mu_{sd}\right)}^{2},$$

where $\mu_{sd}$ is the mean area of the upper-half bounding box over the current time window.

#### 3.2.5. Temporal Variation in the Geometric Center Position

The geometric location of a foreground blob changes significantly when a person falls. This change in geometric location can be captured by taking the temporal variation of geometric center (G.C.) as a feature. The temporal variation in the *x*-component and *y*-component of the geometric center of a current frame is computed by taking the standard deviation of the *x*-component and *y*-component of the previous k=30 frames. The geometric center, $\left(x_{g},y_{g}\right)$, is the average of the *x*-coordinates and *y*-coordinates of all boundary points (edge points) of the object

(6) $$\left(x_{g},y_{g}\right)=\frac{1}{n}\left(\sum_{i=1}^{n}x_{i},\sum_{i=1}^{n}y_{i}\right),$$

where $\left(x_{i},y_{i}\right)$ represents the coordinates of the pixels in the foreground blob. Figure 2 shows the temporal variation of the geometric center of a falling person. In this figure, the number of frames and geometric center are plotted along the *x*-axis and *y*-axis, respectively.

(7) $$\sigma_{gp}^{2}=\frac{1}{k}\sum_{i=1}^{k}{\left(g_{p}\left(i\right)-\mu_{gp}\right)}^{2},$$

where $\mu_{gp}$ is the mean position of the geometric center over the current time window.

#### 3.2.6. Temporal Variation of the Ellipse Ratio

The ellipse ratio, $e_{r}$, is defined as the ratio between the length of the major and minor axes of the ellipse containing the foreground blob. It was used as a scale invariant feature in our proposed approach for the classification of a person as fall or not a fall. Figure 1A,B shows that the ellipse ratio changes significantly when the person is moving from a standing state (A (a) & B (a)) to intermediate states (A (b), B (b) and A (c), B (c)) and then to the fall state ( A (d) & B (d)). Hence, the ratio of these axes of ellipse serves as an important feature for detecting the human fall. The temporal variation of ellipse ratio is computed over the current time window

(8) $$\sigma_{er}^{2}=\frac{1}{k}\sum_{i=1}^{k}{\left(e_{r}\left(i\right)-\mu_{er}\right)}^{2},$$

where $\mu_{er}$ is the mean ellipse ratio over the current time window.

Figure 1 shows a comparison of different proposed feature values for two different camera views. The range of feature values despite significant view changes remained almost the same. Figure 2 shows a comparison of the temporal variation of different features in two different views. Despite significant variations in the camera viewing angle, the shape of the temporal variation remained almost the same. Both figures show that the feature values and temporal variation of values remained almost unchanged regardless of the camera viewing angle. This is the main reason for the consistent performance of the proposed algorithm across multiple camera views.

Similarly, a different camera view is shown in Figure 2. The behaviour of our features is equally good in this new figure with different camera views; this shows that our features exhibit comparable performance even with different views.

### 3.3. Training Boosted J48 Classifier

Classification is the task of finding a target function that maps an attribute set to a particular class among a predefined set of classes. Various classifiers including SVM, neural networks, rule-based methods, prototype methods and exemplar-based methods exist in the literature. In the current work, we trained an ensemble of J48 classifiers, which we named ‘Boosted J48’. We prefer this classification strategy over the other existing classifiers mainly because of its speed and accuracy.

Boosting is a method for combining multiple classifiers [42,43]. As the name suggests, it is a meta algorithm that is used to improve the results of the base classifier. In our case, the base classifier was J48 which is an extension of the ID3 algorithm that generates rules for the prediction of target variables [44]. The additional features of J48 include finding missing values, derivation of rules, continuous attribute value ranges, and decision tree pruning.

Boosting works sequentially, whereby the first algorithm is trained on the entire dataset, and then the rest of the algorithms are developed by fitting the residuals of the first algorithm. In this process, higher weight is given to the observations that have been poorly predicted by the previous model. Boosting is known to be sensitive to noisy data and outliers. The reason for this is that boosting overfits noisy datasets. Boosting on stable algorithms like J48 improves performance, while boosting on unstable algorithms, like MLP, may reduce performance [45].

In each iteration, the base classifier is used with a different weight over the samples of the training set. At each iteration, the computed distributions assign more weight to the incorrectly classified samples. The final classifier is obtained as a weighted average of the previous hierarchical designed classifier. We focused on the two-class classification task, where the training set is $\left\{\left(x_{1},y_{1}\right),\left(x_{2},y_{2}\right),\ldots ,\left(x_{N},y_{N}\right)\right\}$, $x_{i}$ is some feature vector and $y_{i}\in \left\{-1,1\right\},i=1,2,\ldots ,N$ is the label. The aim was to design an optimal classifier to predict the label of a test feature vector, $x_{t}$.

(9) $$\hat{y}=\mathrm{sign}\left\{F\left(x_{t}\right)\right\}$$

where $\hat{y}$ is the predicted label and

(10) $$F\left(x_{t}\right)=\sum_{k=1}^{K}\propto_{k}\phi \left(x_{t};\theta_{k}\right),$$

where $\phi (x_{t};\theta_{k})$ denotes the base classifier that returns a binary class label, {−1,1}. The corresponding parameter vector, $\theta_{k}$, describes the base classifier. An important property of boosting is its relative immunity to overfitting with an increasing *K*. It has been verified that even with a high number of terms, *K*, and consequently, a high number of parameters, the error rate on a test set does not increase but keeps decreasing and finally, reaches a low, asymptotic value.

Random Decision Forests (RDF) and J48 are both tree-based classifiers. RDF is a mixture of tree predictors, where each tree is a predicate of the values of a random vector sampled autonomously and all the trees of the forest have the same distribution [46]. As the trees in a forest become large in number, the generalization error for the forest converges to a certain limit. The votes from all trees determine the class assignments. The main limitation of RDF is its increased complexity with an increasing depth of trees when training RDF, compared to J48. That is, RDF requires more computational resources and has higher memory complexity. The learning rate of RDF is slower and its prediction process also has more computational complexity compared to an equivalent J48 classifier.

As discussed, J48 is an extension of the ID3 algorithm that generates rules for the prediction of target variables [44]. The additional features of J48 include finding missing values, the derivation of rules, continuous attribute value ranges, decision tree pruning, etc. J48 is based on the information gain ratio that is evaluated by entropy. The information gain ratio measure is used to choose the test features (attributes) at each node in the tree. The attribute with the highest information gain ratio is selected as the test feature for the current node. If we have a feature, X, and we examine the values for this feature in the training set and they are in increasing order, $A_{1},A_{2},\ldots ,A_{m}$, then for each value, $A_{j}$, j=1,2,…,m, the records are partitioned into 2 sets: the first set includes the X values up to and including $A_{j}$ and the second set includes the X values greater than $A_{j}$ [47]. For each of these m partitions, the GainRatio(X(j),T) where j=1,2,…,m is computed, and the partition that maximizes the gain is chosen. The GainRatio(X,T) is given in Equation (11):

(11) $$GainRatio\left(X,T\right)=\frac{Gain(X,T)}{SplitInfo(X,T).}$$

Considering the information content of a message that indicates not the class to which the case belongs, but the outcome of the test on feature X, the SplitInfo is given by Equation (12):

(12) $$SplitInfo\left(X,T\right)=-\sum_{i}^{n}\frac{|T_{i}|}{\left|T\right|}log_{2}\frac{|T_{i}|}{|T|.}$$

The GainRatio(*X*,*T*) is thus the proportion of information generated by the split that is useful for the classification.

## 4. Experiments and Results

### 4.1. Datasets

We performed experiments on two publicly available datasets: the Multiple Cameras Fall (MCF) dataset (http://www.iro.umontreal.ca/~labimage/Dataset/) and the UR Fall Detection (URFD) dataset (http://fenix.univ.rzeszow.pl/~mkepski/ds/uf.html). Table 1 contains the descriptions of 20 videos from the MCF dataset captured at camera position 2. The details mentioned in Table 1 include the video number, total frames in each video, frames with falls and frames without falls. Figure 1 shows some example frames from the MCF dataset from video 1 (camera 7) and video 3 (camera 3) respectively containing various states of a falling person.

**Figure 3:**
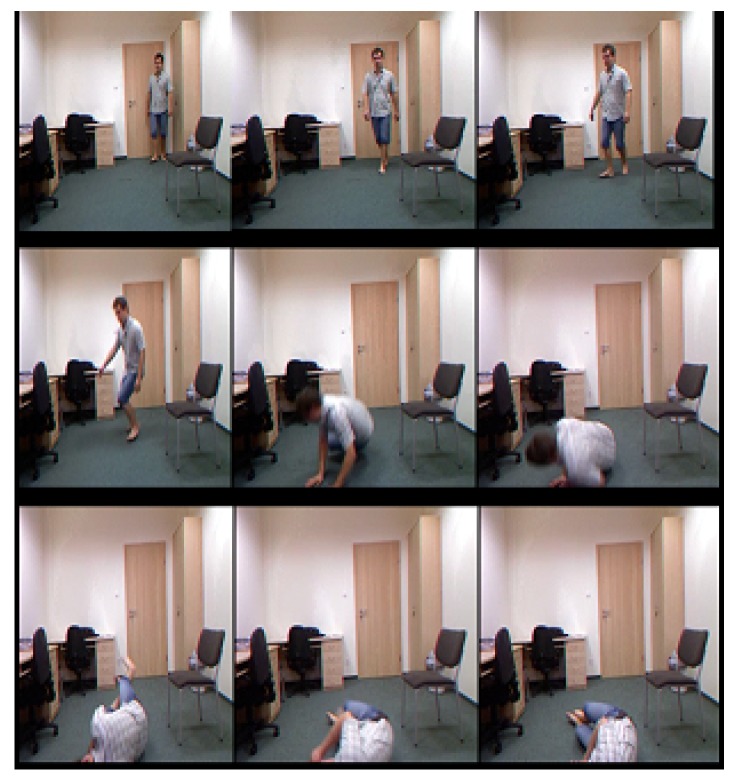
Selected frames from the frontal sequence of the URFD dataset.

The UR Fall Detection (URFD) dataset contains frontal and overhead video sequences obtained by two Kinect sensors, with one placed at the height of 1 m from the floor and the other mounted on the ceiling with a height of 3 m. The dataset contains two kinds of falls that were performed by five people—from standing position and from sitting on the chair. The dataset was recorded at 30 frames per second. The frontal sequence contains 314 frames, in which 74 frames contain falls and 240 frames have no falls. The key frames of the URFD dataset are shown in Figure 3. The overhead sequence contains a total of 302 frames, in which 75 frames contain falls, while 227 have no falls.

In all experiments, 10-fold cross-validation was used. For the purpose of temporal variance computation in Equations (1)–(8), different temporal window values, *k*, were used. All results reported in the paper are for *k* = 30. Since the video frame rate was 30 fps, *k* = 30 corresponds to a duration of 1 s.

### 4.2. Comparison with Existing Approaches

The proposed algorithm was compared with existing approaches including Kepski et al. [38] using KNN and SVM, Debard (De) [26], Debard Kyrkou (DeKy) [26,30], Debard Foroughi (DeFo) [26,48], Osuna (Osu) [27], Kyrkou (Ky) [27,30] and Foroughi (Fo) [27,48].

The proposed approach, ’PA_B-J48’, uses the temporal variance of motion and geometric features with an ensemble learning strategy of ‘boosting with J48’. The second proposed approach, ‘PA_Ada’, uses the same set of features but with the Adaptive Boosting (AdaBoost) classifier. AdaBoost is an ensemble learning approach based on game theory with a main aim of combining many weak classifiers to produce a strong classifier.

Adaboost is an iterative algorithm that is used in conjunction with many other types of learning algorithms (weak learners) to improve their performance [49]. The output of weak learning algorithms is combined into a weighted sum that forms the final output of the boosted classifier. Adaboost, short for adaptive boosting, tweaks the weak learners in favor of misclassified instances by the previous classifiers. The prior knowledge of the lower bound of prediction accuracy of weak learning algorithms is not required; hence, Adaboost is suitable for many practical purposes. The algorithm is sensitive to noisy data and outliers.

The proposed algorithms work better than deep learning due to various reasons. Deep networks require very large training datasets containing millions of images to achieve good performances. Due to the limited training data being used in this study, our proposed algorithms were more suitable than the deep learning approaches.

Deep networks are computationally expensive as they require high-end GPUs to be trained on large datasets. Expensive GPUs, fast CPU, SSD storage and large RAM significantly increase the hardware cost and computational complexity of deep networks. Hence, it is not feasible to train these deep networks are not feasible on the current systems (64-bit machine with Intel core i3-3110M CPU @2.40GHz, and 4GB RAM) on which our proposed algorithm gives more than 99% percentage accuracy for various data sets.

Classical machine learning algorithms are easier to interpret and understand compared to deep learning methods. Due to thorough understanding of data and algorithms, it is easy to tune hyper-parameters and to change the design of a model. Deep learning networks have often been used like a “black box”.

### 4.3. Performance Measures

In addition to accuracy, we used sensitivity and specificity for a comparison of our system with existing systems . These measures have also used by other fall detection systems [50].

(13) $$Sensitivity=\frac{TP}{\left(TP+FN\right)}$$

(14) $$Specificity=\frac{TP}{\left(TN+FP\right)}$$

where true positive (TP) is the number of falls correctly identified by the system, and false negative (FN) is the number of falls missed by the system, and true negative (TN) is the number of ‘no falls’ correctly identified by the system, and false positive (FP) is the number of ‘no falls’ missed by the system.

### 4.4. Experiments on the MFC Dataset

The performance of the proposed fall detection system was evaluated on the MFC dataset in three different experiments and compared with the existing state-of-the-art approaches. These experiments are discussed below.

#### 4.4.1. Experiment 1

**Figure 4:**
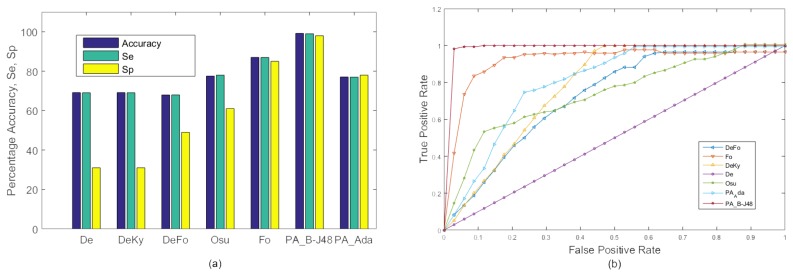
Experiment 1: A comparison of the existing approaches and the proposed approaches on the MCF dataset with 10-fold cross validation using two categories: ‘Fall’ and ‘No Fall’. (**a**) Graph; (**b**) ROC curve.

In the first experiment, frames of all 20 videos were combined and 10-fold cross validation was used to train and test the proposed system. All test frames were classified as fall or no fall. The resulting accuracy, sensitivity, and specificity of the existing as well as proposed methods are shown in Figure 4. The proposed PA_B-J48 has previously been shown to obtain larger accuracy than all compared methods. The proposed PA_Ada and the existing algorithms were not able to efficiently handle within class variations. A possible reason for the accuracy degradation of PA_Ada is overfitting [51]. Adaptive boosting uses a training set over and over and hence, is more prone to overfitting.

Figure 4 shows the ROC curves of the existing approaches, including De, DeKy, DeFo, Osu, Ky, Fo and proposed approaches ‘PA_B-J48’ and ‘PA_Ada’ on MCF dataset (Experiment 1). It may be observed that ROC curve of the proposed approach PA_B-J48 is better than the existing approaches.

Table 2 shows the percentage accuracy of existing approaches, including De, DeKy, DeFo, Osu, Ky, Fo, and the proposed approaches ‘PA_B-J48’ and ‘PA_Ada’ from camera position 3 and video 3 of the MCF dataset (Experiment 1). Camera 3 was placed exactly on the opposite wall from camera 2 (http://www.iro.umontreal.ca/~labimage/Dataset/). It may be observed that the proposed approach ‘PA_Ada’ exhibited a percentage accuracy of 99.14% with camera position 3 which is comparable with the percentage accuracy shown by camera position 2 (as can be seen in Table 3). Hence, our proposed algorithm performed robustly across different camera positions.

In all experiments, the proposed algorithm performed equally well for different types of falls for various videos in the MCF dataset. We experimentally observed that the performance of our proposed algorithm was not greatly effected by the view variations in this dataset. The falls in these videos can be divided into three categories with respect to the camera angle: falling towards a side or falling towards or away from the camera. In videos 1, 3, 5, 6, 9, 10, 11, 12, 14, 15, 18, 19, and 22, the person is walking from the right to the left of the scene and falls to the side with respect to camera. In all of these videos, the person falls on his chest, while in video 6, the person falls on his back. In videos 9 and 10, the person sits on a sofa before falling. Video 11 is similar to video 9, but the person falls on a sofa rather than sitting on it. In videos 12 and 14, the person is crouching or picking up stuff from the ground before falling. In videos 15, 18, and 19, the sofa is replaced by a chair. In video 22, the person slips to the side.

The second type of fall occurs in videos 2, 4, 7, 8, and 13; the person moves from the right to the left of the scene and falls towards the camera. In video 13, the person falls on a sofa as well. In video 4, the person falls twice. The third type of fall occurs in videos 16, 17 and 20; the fall occurs away from the camera. In video 16, the person moves in a circular motion around the sofa and then falls on the sofa away from the camera. In video 20 , the person moves from the left to the right and then from the right to the left multiple times, picking up stuff from the floor, falling on a sofa multiple times and then falling on the floor in a direction away from the camera. The proposed algorithm performed almost the same on all these fall variations.

#### 4.4.2. Experiment 2

**Figure 5:**
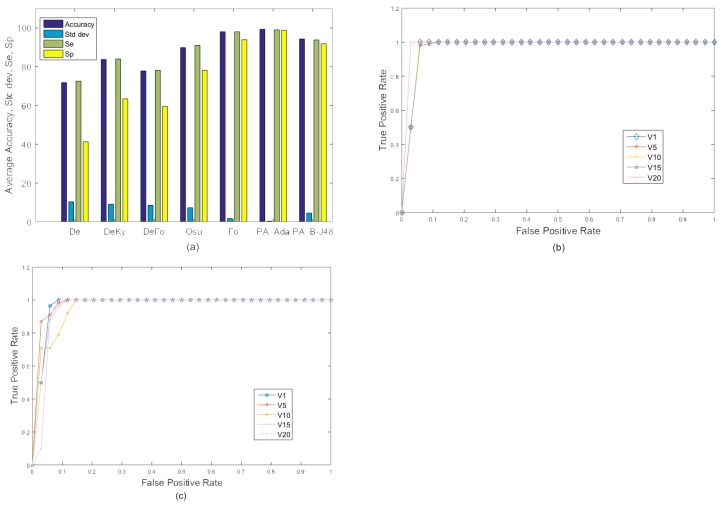
Experiment 2: Average comparison of the existing approaches and the proposed approaches on the MCF dataset with 10-fold cross validation using two categories: ‘Fall’ and ‘No Fall’. (**a**) Graph; (**b**,**c**) ROC curve of selected videos (videos 1, 6, 10, 15, and 20) using camera 2 for the proposed approaches, PA_B-J48 and PA_Ada.

The performance of the existing as well as the proposed algorithms on the MFC data set with 10-fold cross validation is shown in Table 3 and Figure 5. Due to relatively less with-in-class variations, performance of all algorithms increased. In this experiment, both proposed algorithms PA_B-J48 and PA_Ada performed quite well and obtained very high accuracy.

#### 4.4.3. Experiment 3

**Figure 6:**
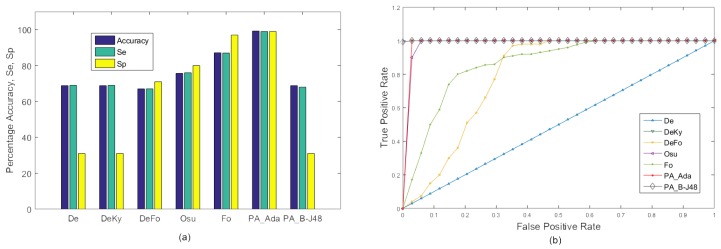
Experiment 3: Accuracy comparison of the existing approaches, De, DeKy, DeFo, Osu, Ky, Fo and the proposed approaches, ’PA_B-J48’ and ’PA_Ada’, on all videos in the MCF dataset with 10-fold cross validation using three classes i.e., ‘fall’, ‘sitting’ and ‘no fall’. (**a**) Graph; (**b**) ROC curve.

In the third experiment, a three-class classification was performed. We added a sitting down class to evaluate the performance of the proposed algorithm in differentiating falling down from sitting down. The main difference between these two actions is the speed of performing the action. Similar to the first experiment, all 20 videos were merged together to form a single video and 10-fold cross validation was used for training and testing. Sitting down is slow and relatively gradual while falling down is rapid and relatively random. The results of this experiment are shown in Figure 6. In this experiment, the performance of PA_B-J48 remained excellent while all other methods suffered from significant accuracy degradation. This was mainly due to the similarity between the sitting and falling down classes. Moreover, adaptive boosting is more prone to overfitting large datasets and it could also serve as an important reason for the reduced performance of PA_Ada [51]. The reason for the better performance of PA_B-J48 is due to an improvement in the efficiency of the basic J48 algorithm through boosting, an ensemble learning method and more effective feature set. The J48 is a powerful decision tree method that can handle both discrete and continuous attributes. The algorithm also handles the missing values in the training data.

### 4.5. Experiments on the URFD Dataset

**Figure 7:**
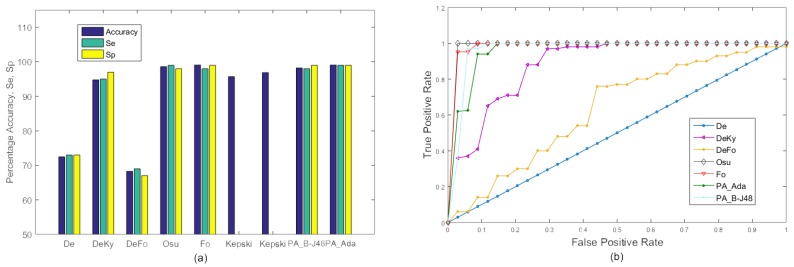
Experiments on the URFD dataset: comparison of accuracy, Se and Sp between the existing approaches under consideration and the proposed approach on the frontal and overhead data sequence from the URFD dataset. (**a**) Graph; (**b**) ROC curve.

The URFD dataset is challenging as it contains abrupt human falls and short video sequences. Moreover, the data set contains falls not only from the standing position, but also while sitting on a chair. It can be seen in the Figure 7 that our proposed approach ‘PA_B-J48’ outperformed the existing approaches of De, DeKy, DeFo, Osu, Fo, and Kepski et al. using KNN and SVM [38] on both frontal and overhead data sequences in terms of average accuracy, sensitivity, and specificity. Moreover, our second proposed approach also exhibited better percentage accuracy than the existing approaches of De, DeKy, DeFo, Fo, and Kepski et al. using KNN and SVM [38].

The proposed algorithm exhibited an accuracy of 99.13% on the frontal video sequence of URFD while it showed an accuracy of 99.03% of the overhead video sequence of the same dataset. This shows that accuracy degradation occurs despite significant view variation being quite minor.

### 4.6. Execution Time Comparison

A comparison of the execution times of the proposed approaches, PA_B-J48 and PA_Ada, with the existing approaches was performed on the MCF dataset (Experiments 1 & 3) and the URFD dataset, as shown in Table 4. All experiments were performed on a 64-bit machine with Intel Core i3-3110M CPU @2.40GHz, and 4GB RAM. In Experiment 1, the proposed PA_Ada was 9.06, 74.28, and 27.29 times faster than DeFo, Osu and Fo, respectively, while the PA_B-J48 method was 0.57, 4.72, and 1.73 times faster, respectively. In Experiment 3, the proposed PA_Ada was 4.28, 37.14, and 16.06 times faster than DeFo, Osu and Fo, respectively, while PA_B-J48 was 0.60, 5.24, and 2.27 times faster, respectively, than these methods. In both of these experiments, PA_Ada remained significantly faster than the compared algorithms. PA_B-J48 remained faster than both Osu and Fo. In both experiments, DeFo remained faster than PA_B-J48; however, its accuracy remained low. Similarly De and DeKy were faster but significantly less accurate. This shows a trade-off between accuracy and speed. The proposed algorithm uses a larger feature set than these methods to achieve a higher accuracy. By using a parallel implementation of the proposed PA_B-J48, its execution time can be significantly reduced over larger datasets. On smaller datasets such as URFD, the proposed PA_B-J48 is already among the fastest methods (Table 4).

## 5. Conclusions

An accurate and fast human fall detection system is of utmost importance for patients and aged persons for timely intervention if a fall happens, to avoid serious injuries or consequences from a fall. The work presented in this paper used the temporal variance of various discriminatory features including motion, geometric orientation and geometric location to build a fall detection system. The proposed system was trained and tested using the ensemble learning strategy and boosting with the J48 classifier as well as with the AdaBoost classifier. The proposed system was tested on two publicly available fall detection datasets and compared with eight existing algorithms. From the experiments, it was concluded that the accuracy of the proposed system was better than existing approaches for both the datasets. The proposed system was better able to differentiate between sitting down and falling down compared to existing algorithms. The proposed system also offers a faster execution time than existing methods.

This work can be extended to further improve the performance and time efficiency of human fall detection systems, specifically in more challenging datasets containing multiple people with occlusions, the same colored clothes as that of background and multisource, non-Lambertian lighting. In addition, the proposal of more novel and robust features and the development of large video repositories will play vital roles in improving the accuracy and robustness of fall detection systems. The addition of night vision functionality to a system will be an important feature of outdoor fall detection systems.

## Figures and Tables

**Table 1:** Details of the Multiple Cameras Fall (MCF) Dataset: total number of frames, number of frames containing a fall, and number of frames containing no fall in each video.

Videos	Total Frames	Fall	No Fall	Videos	Total Frames	Fall	No Fall
Video 1	1411	314	1097	Video 11	1486	608	878
Video 2	756	331	425	Video 12	1041	420	621
Video 3	883	272	611	Video 13	1240	360	880
Video 4	1033	409	624	Video 14	970	385	585
Video 5	600	266	334	Video 15	1007	367	640
Video 6	1203	513	690	Video 16	1023	320	703
Video 7	912	290	622	Video 17	992	432	560
Video 8	700	240	460	Video 18	1217	627	590
Video 9	905	360	545	Video 19	1155	390	765
Video 10	813	230	583	Video 20	2328	90	2238

**Table 2:** Experiment 1: Comparison of the existing approaches, De, DeKy, DeFo, Osu, Ky, and Fo, with the proposed approaches ‘PA_B-J48’ and ‘PA_Ada’ in terms of % accuracy (A) at camera position 3 of video 3 (V) of the MCF dataset with 10-fold cross validation using two categories: ‘Fall’ and ‘No Fall’. Maximum value in each row is shown in bold.

Existing Approaches							Proposed Approaches	
		De [26]	DeKy [30]	DeFo [48]	Osu [27]	Fo [48]	PA_B-J48	PA_Ada
Cam3 (V3)	A	73.23	77.09	78.37	93.47	98.39	92.4	**99.14**

**Table 3:** Experiment 2: Comparison of the existing approaches, De, DeKy, DeFo, Osu, Ky, and Fo, with the proposed approaches, ‘PA_B-J48’ and ‘PA_Ada’, in terms of % accuracy (A), % sensitivity (Se) and % specificity (Sp) for each Video (V) of the MCF dataset with 10-fold cross validation using two categories: ‘Fall’ and ‘No Fall’. Maximum value in each row is shown in bold.

Existing Approaches							Proposed Approaches	
Video		De [26]	DeKy [30]	DeFo [48]	Osu [27]	Fo [48]	PA_B-J48	PA_Ada
V1	A	88.65	93.76	90.50	99.23	**99.86**	99.65	98.99
	Se	89.00	94.00	91.00	98.00	**99.00**	**99.00**	**99.00**
	Sp	69.00	95.00	86.00	97.00	**99.00**	**99.00**	**99.00**
V2	A	61.83	91.76	71.24	93.03	**99.74**	99.08	99.34
	Se	62.00	92.00	71.00	98.00	**99.00**	**99.00**	99.00
	Sp	49.00	85.00	61.00	97.00	**99.00**	**99.00**	**99.00**
V3	A	72.79	92.86	74.94	99.88	99.89	99.89	**100.00**
	Se	73.00	93.00	75.00	99.00	99.00	99.00	**100.00**
	Sp	42.00	91.00	71.00	99.00	99.00	99.00	**100.00**
V4	A	68.41	82.95	70.45	87.40	98.35	**99.42**	88.85
	Se	68.00	83.00	70.00	87.00	98.00	**99.00**	89.00
	Sp	31.00	55.00	64.00	77.00	98.00	**99.00**	70.00
V5	A	63.42	89.38	90.12	92.03	95.72	**98.82**	94.99
	Se	63.00	89.00	90.00	92.00	96.00	**99.00**	95.00
	Sp	65.00	98.00	98.00	87.00	93.00	**99.00**	98.00
V6	A	91.51	89.33	94.01	95.34	99.58	**99.67**	98.17
	Se	91.00	90.00	94.00	95.00	**100.00**	99.00	98.00
	Sp	85.00	89.00	87.00	91.00	**100.00**	99.00	98.00
V7	A	68.09	85.42	79.17	95.39	99.23	**99.45**	98.90
	Se	68.00	85.00	79.00	95.00	**99.00**	**99.00**	98.00
	Sp	32.00	62.00	76.00	89.00	**99.00**	**99.00**	98.00
V8	A	65.57	83.86	72.57	89.14	99.00	**99.29**	98.42
	Se	66.00	84.00	73.00	89.00	**99.00**	**99.00**	98.00
	Sp	32.00	61.00	78.00	72.00	98.00	**99.00**	98.00
V9	A	70.50	85.19	77.46	91.60	97.13	**99.45**	96.46
	Se	71.00	85.00	78.00	92.00	97.00	**99.00**	97.00
	Sp	52.00	90.00	74.00	97.00	95.00	**99.00**	76.00
V10	A	77.74	80.93	80.07	91.39	98.40	**99.02**	95.69
	Se	78.00	81.00	81.00	91.00	98.00	**99.00**	96.00
	Sp	65.00	52.00	52.00	84.00	94.00	**99.00**	93.00
V11	A	59.02	59.02	65.75	84.86	98.92	**99.53**	87.35
	Se	59.00	59.00	66.00	85.00	**99.00**	**99.00**	87.00
	Sp	41.00	70.00	55.00	84.00	**99.00**	**99.00**	95.00
V12	A	63.01	83.19	74.54	93.28	98.85	**99.33**	95.58
	Se	79.00	79.00	79.00	93.00	**99.00**	**99.00**	96.00
	Sp	21.00	21.00	21.00	89.00	**99.00**	**99.00**	97.00
V13	A	78.93	78.93	78.93	78.93	95.24	**99.11**	89.26
	Se	79.00	79.00	79.00	93.00	95.00	**99.00**	89.00
	Sp	21.00	21.00	21.00	6.00	84.00	**97.00**	74.00
V14	A	64.08	64.29	68.84	79.81	96.58	**98.96**	89.64
	Se	64.00	64.00	68.00	80.00	97.00	**99.00**	90.00
	Sp	36.00	39.00	51.00	57.00	95.00	**98.00**	92.00
V15	A	63.72	90.85	72.56	91.65	96.02	**99.50**	86.18
	Se	63.00	91.00	73.00	92.00	96.00	**99.00**	86.00
	Sp	40.00	70.00	72.00	96.00	95.00	**99.00**	87.00
V16	A	69.60	85.92	69.60	74.19	94.33	**99.41**	89.34
	Se	79.00	86.00	70.00	74.00	94.00	**99.00**	75.00
	Sp	30.00	67.00	30.00	42.00	90.00	**99.00**	89.00
V17	A	72.53	83.13	75.46	78.30	97.46	**98.83**	93.26
	Se	72.00	81.00	76.00	78.00	98.00	**99.00**	93.00
	Sp	27.00	70.00	47.00	49.00	93.00	**98.00**	86.00
V18	A	65.24	79.79	78.14	90.30	99.06	**99.18**	92.76
	Se	65.00	90.00	78.00	98.00	**99.00**	**99.00**	**99.00**
	Sp	37.00	72.00	88.00	99.00	99.00	99.00	**100.00**
V19	A	76.19	78.79	77.06	93.42	97.84	**99.48**	93.42
	Se	76.00	79.00	77.00	93.00	**99.00**	**99.00**	93.00
	Sp	48.00	52.00	56.00	88.00	55.00	**99.00**	92.00
V20	A	96.09	96.09	96.06	98.58	**99.87**	**99.87**	99.83
	Se	96.00	96.00	96.00	99.00	**100.00**	**100.00**	**100.00**
	Sp	3	3	3	65.00	96.00	**99.00**	96.00

**Table 4:** Comparison of execution times (seconds) of the existing approaches under consideration and the proposed approaches on the MCF dataset (Experiments 1 & 3) and on the URFD dataset. The execution time was measured on the same machine under similar operating conditions.

Existing Approach				Proposed Approach	
Experiment	DeFo	Osu	Fo	PA_B-J48	PA_Ada
Experiment 1	16.5 s	135.19 s	49.65 s	28.63 s	1.82 s
Experiment 3	13.05 s	113.28 s	48.98 s	21.6 s	3.05 s
URFD (average)	0.12 s	0.01 s	0.32 s	0.01 s	0.025 s
